# Modeling & implementation of DRLA based partially shaded solar system integration with 3-*ϕ* conventional grid using constant current controller

**DOI:** 10.1016/j.heliyon.2022.e09669

**Published:** 2022-06-06

**Authors:** Radhika Guntupalli, M. Sudhakaran, P. Ajay-D-Vimal raj

**Affiliations:** Department of EEE, Pondicherry technological university, Puducherry, India

**Keywords:** Maximum power point tracking (MPPT), Deep reinforcement learning algorithm (DRLA), Deep learning algorithm (DLA), Reinforcement learning algorithm (RLA), Deep deterministic policy gradient (DDPG), Partial shading conditions (PSC), Pulse width modulated voltage source inverter (PWM-VSI), Machine learning (ML), Standard test conditions (STC)

## Abstract

Renewable Energy Resources (RERs) are widely used on the concern of global environment protection. Solar energy systems play an important role in the generation of electrical energy, remarkably minimize the utilization of nonrenewable fuel sources. Solar energy can be extracted and transformed into electrical energy via solar photovoltaic process. Several traditional, soft computing, heuristic, and meta-heuristic maximum power point tracking (MPPT) techniques have been developed to extract Maximum Energy Point (MEP) from the solar photovoltaic modules under different atmospheric conditions. In this manuscript, the combination of reinforcement learning algorithm (RLA) and deep learning algorithm (DLA) called deep Reinforcement Learning Algorithm based MPPT (DRLAMPPT) is proposed under partial shading conditions (PSC) of the solar system. DRLAMPPT can deal with continuous state spaces, in contrast to RL it can be operated only with discrete action state spaces. In this proposed DRLAMPPT, deep deterministic policy gradient (DDPG) solves the problem of continuous state spaces are involved to reach the GMEP in photovoltaic systems especially under PSC. In DRLAMPPT, the representative's strategy is parameterized by an artificial neural network (ANN), which uses sensory information as input and directly sends out control signals. This work develops a 2 kW solar photovoltaic power plant comprises of a photovoltaic array, DC/DC step-up converter, 3-Φ Pulse Width Modulated Voltage Source Inverter (PWM-VSI) integrated with conventional power grid using Constant Current Controller (CCC Effectiveness of the proposed DRLAMPPT with CCC can be validated through an experimental setup and with MATLAB. Simulation and tested at different input conditions of solar irradiance. Experimental results prove that, in comparison to existing MPPTs, suggested DRLAMPPT not only attains the best efficiency and also adopts the change in environmental conditions of the photovoltaic system at a much faster rate and able to reach the GMEP within 0.8 s under PSC. Experimental and simulation results also prove that suggested CCC with LC filter makes the inverter output voltage and the grid voltage are in phase at the lower value of THD i.e. 1.1% and 0.98% respectively.

## 1 Introduction

The demand for electrical energy continues to grow and is expected to increase substantially hereafter [1], drives the fast development of RERs such as solar, wind, tidal, geothermal, etc. results in lower fossil fuel consumption and environmental protection. Like wind energy, solar power is the most considerable source of energy and has a significant market share in the world electrical industry. Due to the steady decline in prices and growing concern about carbon emissions, photovoltaic systems are densely built in regions with larger irradiation [2]. The sun radiation and temperature acting on the PV modules varies depending on the latitude, direction of the sun, season, and time of the day. Although photovoltaic cells produce clean energy and the sun is considered an inexhaustible source of energy, they have two main disadvantages: one is the low conversion efficiency of solar irradiance, and the other is nonlinear power-voltage (PV) & current-voltage (IV) characteristics add additional complexity due to different atmospheric conditions (temperature and light) [3]. Design and the implementation of the MPPT controller allow PV power sources to work in MPP at any time under different atmospheric conditions, which is essential for maximizing the efficiency of photovoltaic modules [4], [5]. To enhance the performance of the photovoltaic module and to make the system performance better, it is essential to develop effective MPPT techniques. Many MPPT technologies have been used over the years and can be classified based on reliability, responsiveness, efficiency, and memory.

The prominent technologies are Perturb and Observation (P&O) and incremental conductance (INC) [6], [7]. These traditional MPPT algorithms [5] have been practically applied because of their accessibility and ease of development. In addition, many other traditional algorithms were proposed [9]. These methods can effectively work only under uniform sunlight conditions [8], [10]. In traditional MPPT algorithms getting stuck to a local MPP, which leads to the low energy conversion under PSC is a considerable disadvantage. Modified P&O [11] with a variable step size is suggested to eradicate the disadvantages of poor tracking convergence and large oscillations around MEP [12]. In modified P&O, it selects a larger step size if the MEP is still far away and step size is reduced when it is closer. Other types of MPPT algorithms [13], [14], [15], [16], [17] are derived from computational intelligence, such as fuzzy logic based MPPT (FLCMPPT) [13], ANN [14], neuro-fuzzy [16], etc. AIMPPT techniques possess convergence towards GMEP under PSCs within less time of computation, but the execution is very difficult and also requires high-cost microcontrollers. Few other types of stochastic MPPT algorithms are derived from biological process those are genetic algorithm (GA) [17], cuckoo search (CS) [18], ant colony optimization (ACO) [19], bee colony algorithm (BCA) [20], and bio-inspired memetic salp swarm algorithm [20], etc. can deal with the nonlinearities of solar PV system and able to reach the GMEP under PSCs. However, they have two major drawbacks, generally requires an expensive microprocessor for less computation time and knowledge of a specific PV system for less randomness of convergence. Different types of MPPT techniques encapsulated comprehensively [44], they are divided into seven groups, e.g. traditional techniques, meta-heuristic techniques, hybrid techniques, AI based techniques, techniques based on characteristic curves, and other algorithms [33], [34], [35], [36], [37], [38], [39], [40]. Specifically, there are 25 metaheuristic MPPT techniques which are divided into three categories namely, biology-based techniques, physics-based techniques, and sociology-based techniques [44]. Few types of meta-heuristic MPPT controllers [45] are derived from evolutionary techniques [42] and physical process such as particle swarm optimization (PSO) [43], simulated annealing [46], gravitational search algorithm [47], firework algorithm [48], mine blast algorithm [49], and wind driven optimization [50] etc. These methods effectively trace and greatly reduce the oscillations around the GMEP under PSCs. However they have two major disadvantages; high-cost processors are required to achieve fast convergence and random behavior under transient conditions, which will negatively affect the convergence time and system performance. Other classes of MPPT control techniques are hybrid techniques [44] such as Whale optimization and differential evolution (WADE) [51], directive adaptive neural network (DANC) [52], Dynamic leader based collective intelligence [53] etc. attain GMEP effectively under PSCs of solar PV system. However, their design, structure is very much complex and the computational effort is therefore much greater for each individual. Dynamic leader based collective intelligence (DLCI) consists of several sub-optimizers that can achieve much broader investigation by fully cooperating with the optimization ability of multiple search operation instead of a single search operation. To attain the deeper exploitation, the sub-optimizer with the best solution is selected as dynamic leader for an effective searching mechanism to remaining sub-optimizers. But multiple sub-optimizers in DLCI will result greater computational complexity. The selection of the appropriate algorithms for the combination remains not answered. Most of the present stochastic optimized MPPT algorithms are planned with a unique searching mechanism leads to weak searching capability and can trap to local MEP instead of GMEP under PSCs. Existing stochastic optimized MPPTs are designed with arbitrary searching mechanism may lead to different optimal values under same weather conditions. A Fast GMPPT scheme Based on Collaborative Swarm Algorithm (CSA) is proposed [54] to handle PSC based PV system, this method has simple arrangement and requires only two parameters to tune the network. This proposed CSA can track the GMEP under PSC within very short time. A new global MPPT technique using improved PS-FW algorithm for PV system under PSC is proposed [55]. The combination of PSO and FWA along with adaptive control of parameters was provided to balance the exploration and exploitation capabilities; as well as this hybrid algorithm conducts great local search to trace the single GMEP under change in environmental conditions. By introducing an adaptive control, it is possible to control the explosion sparks generated in the last steps of the algorithm. This hybrid algorithm provides excellent tracking convergence and efficiency under PSCs of PV systems in comparison with PSO and FWA alone.

Recent time considerable studies have been done on Reinforcement learning (RL) with successful applications due to its exceptional learning capability and do not require complex mathematical models, so system recognition is not required but can construct a control loop based on interaction with the real photovoltaic environment or a set of trajectories derived from simulation. RL has higher convergence in less computation time compared to meta-heuristic methods. In RLMPPT, maximize the cumulative of future returns, where the return is the benefit given to the representative by the environment after each interaction, showing how well the actions related to the goal are performed [21]. The RL model is an unsupervised learning environment in which artificial agents (representatives) continue to learn and behave directly based on rough interactions with the environment (usually called policies); i.e., with the PV system. RL based MPPT technology has been proposed both for uniform radiation [22], [23] and PSC [24], [25]. To reduce the effort of RLMPPT techniques, frequently the action space has got to be discretized. The RLMPPT algorithm, which relies only on 4 states and 4 actions is proposed [26], these actions are derived as per the direction of the movement towards MEP. However, to improve the performance of MPPT, it is very much necessary to work with continuous state spaces. The main disadvantage of RLMPPT is the use of small discrete-action state spaces and complications in the formulation of RL lie in the action space and static state, so a function approximator is necessary to evaluate control strategies and value functions.

A typical artificial neural network (ANN) based controller i.e. ANFIS [53] is suggested to solve the problem associated with tracking of GMEP under non uniform solar irradiance and temperature conditions. It is a supervised data learning methodology which uses fuzzy to convert PV system inputs (irradiance and temperature) into required outputs with the use of huge mutually dependent NNs. This controller integrates the advantages of two artificial intelligence methodologies into a unique methodology. This controller is simple to implement and can achieve better average efficiency of 97%, as well as it can trace the GMEP of solar system under various climatic conditions with the same flexibility. It is a model free algorithm and needs prior knowledge of the PV system, so it is a bit more memory intensive and needs more space. It also has the fundamental drawbacks of FL, where the skilled knowledge is required to design the FL rules and functions.

The latest development of ML leads to combination of RL and DL, the so-called deep reinforcement learning (DRL) [31], [32], which is considered a significant and potential tool for solving the problem of MPPT optimization with huge state and action spaces. In this manuscript, a model-free deep learning algorithm or DRLA is proposed to solve the difficulties involved in MPPT control of photovoltaic arrangement under random atmospheric conditions i.e. PSC. DRLAMPPT is a model free and dynamic methodology to work with the complicated systems. DRLA can be considered as advancement technology of RL can handle with the large discrete actions, states and spaces. This DRLA relies upon the DDPG method and utilizes ANN to parameterize the strategies and deals with continuous state space actions. The state of a representative is described by sensing elements, without any preprocessing. Whereas the continual actions chosen by the neutral representative corresponds to the command actions for MPPT. Conversely, suggested DDPG based DRLAMPPT can apply multiple searching mechanisms simultaneously, so that a deeper and broader global search can be performed. This dynamic algorithm remarkably reduces the randomness in convergence and attains GMEP in less time of computation.

Mathematical modeling of photovoltaic systems can be done using a single diode model [27] or a two diode model [28]. Both the models have the same ability to extract the unknown parameters. But single diode model is simple, accurate, & requires less computation time. Hence mathematical version of the single diode model of PV array is proposed. In real time applications of solar photovoltaic array integrated with the grid, single-stage [29] and two-stage adoption arrangements [30] may be used. In single stage conversion system, multilevel inverters are used to convert variable DC voltage into AC voltage [31]. In this proposed work two-stage conversion system is used, one DC/DC converter is used to convert variable DC into fixed DC and another power electronic converter is used to invert fixed DC to fixed AC. Two-stage or multiple stage conversion system distributes the control into two separate responsibilities to achieve greater effective energy harvesting. Various current controller strategies are available in the integration of solar PV array with 3-Φ traditional power grid. Mainly current controller strategies can be classified as linear & nonlinear methods. Linear current control techniques are PI (Proportional Integral) [28], PR controller (Proportional Resonant) [29] and RC controller (Repetitive controller) [30]. These controllers can reduce the steady state error, but don't show any effect on the elimination of harmonics. Nonlinear control strategies are Predictive controller, Dead-beat, Hysteresis controller, PSO, GA & Constant Current Controller (CCC).

**Figure 1:**
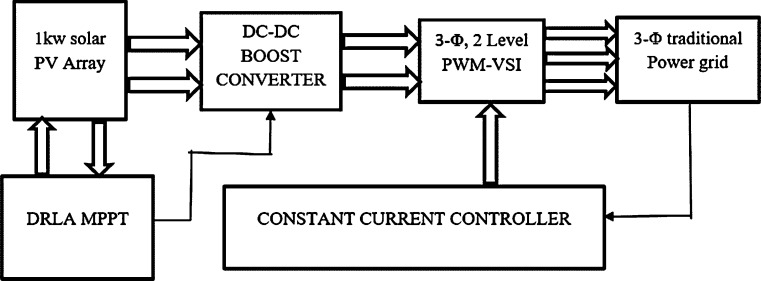
Block diagram of 3-Φ conventional power grid-tied DRLA based partially shaded solar system.

In this work, two-stage energy conversion is proposed which comprises of a photovoltaic array, DC/DC step-up converter, and 3-Φ PWM-VSI integrated with a conventional power grid using Constant Current Controller (CCC) [14]. MEP of the solar PV array can be obtained using DRLA under the conditions of non-uniform irradiance such as PSC. This paper proposes modeling, layout, and hardware implementation DRLA based partially shaded solar system integration with 3-*ϕ* conventional grid using CCC. DC voltage produced by the solar photovoltaic array is increased by using a DC/DC step-up converter. This fixed DC is given as input to the 3-Φ 2 level PWM-VSI. Control of the 3-Φ 2 level PWM-VSI is provided through the CCC. This controller utilizes PLL, to follow the phase angle and conventional power grid voltage. A schematic outline of the suggested work is represented in Fig. 1.

The main contributions of this manuscript are

• Efficient and reliable DDPG based DRLAMPPT is proposed to harvest the MEP under non-uniform solar irradiant conditions is simulated in MATLAB/Simulink environment.
• Three different situations under various climatic conditions are considered to test the effective performance of the DRLAMPPT.
• Modeling, design & hardware implementation of three phase conventional grid connected DRLA based partially shaded PV system with proposed CCC.

In this manuscript, chapter 2 explains the mathematical version of the solar photovoltaic array and the influence of PSC on the position of MEP. DDPG based DRLA, and its mathematical modeling is explained in chapter 3. Description of Constant Current Controller (CCC) is given in chapter 4, Simulation and experimental results of the DRLA based partially shaded solar system integrated with 3-Φ traditional power grid using CCC are explained in chapter 5. Conclusion is given in chapter 6.

## 2 Mathematical version of solar photovoltaic array and the influence of PSC on the location of MEP

### 2.1 Mathematical version of PV array

**Figure 2:**
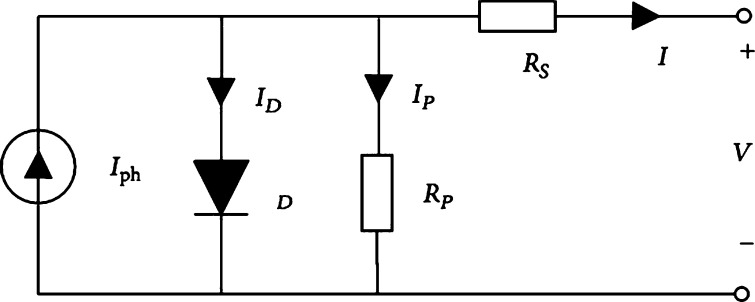
Single diode model of a photovoltaic cell.

Each photovoltaic cell generally has a PN junction and transforms solar irradiation into electrical energy. There are two varieties of photovoltaic models, including dual-diode and single-diode model as shown in Fig. 2. Even though the single-diode model is not as precise as the other one, it is favored for its simplicity. Commercially available silicon cell produces a current between 28 mA/cm^2^ to 35 mA/cm^2^. When cells are connected in series & parallel, then current and voltage ratings can be increased. This group of cells is known as a photovoltaic array. In this proposed work, 8 photovoltaic modules with 250 W are connected in series, the technical data of the photovoltaic modules are given in Table 1.

**Table 1:** Technical specifications of 250 W photovoltaic module.

Type of module	250 W
Maximum power	250 W
Tolerance	±5%
Open circuit voltage	37.25 V
Short circuit current	8.95 A
Maximum Power current	6.25 A
Maximum power voltage	28.5 V
Module efficiency	15.6%
No. of cells in series	60
Solar cell efficiency	18.6%
Series fuse rating	15 A
Type	Multi crystalline

As per the Eq. (1), the output current [41] generated by the photovoltaic cell is given by

(1) $$I=I_{\mathrm{photon}}-I_{d}-I_{P}$$

(2) $$I_{P}=\frac{V+{\mathrm{IR}}_{\mathrm{Se}}}{R_{P}}$$

As per the Eq. (2), $I_{p}$ is the current [41] through the shunt resistance, [41] which is connected in parallel with the diode and $R_{\mathrm{se}}$ is the series resistance.

$I_{\mathrm{ph}}$ is the current produced by each PV cell [41] due to light, and it is proportional to the intensity of light given by Eq. (3).

(3) $$I_{\mathrm{ph}}=[I_{\mathrm{sc}}+K_{1}(T_{C}-T_{\mathrm{ref}})]X\frac{G_{i}}{G_{\mathrm{STC}}}$$

Where $I_{\mathrm{sc}}$ [41] is the current at SCC,

$G_{\mathrm{STC}}$ & *T* are the standard test conditions of irradiance and temperature respectively (T=25°C, $G_{\mathrm{STC}}=1000\text{ W}/{\text{m}}^{2}$),

$T_{\mathrm{ref}}$ is the reference temperature,

$K_{1}$ is temperature coefficient,

$G_{i}$ is relative radiation,

$I_{d}$ is the current flows through the diode given by Eq. (4).

(4) $$I_{d}=I_{0}[\exp\frac{qVd}{\mathrm{AK}T_{C}}-1]$$

Where $q=1.6\times 10^{-19}$

$K=1.38\times 10^{-23}$

$I_{0}$ is reverse saturation current

(5) $$V_{d}=V+{\mathrm{IR}}_{s}$$

$V_{d}$ is voltage drop [41] across the diode given by Eq. (5).

The current produced by each photovoltaic module [41] can be calculated by using Eq. (6)

(6) $$I_{\mathrm{pv}}=I_{\mathrm{ph}}-I_{O}[\exp\frac{q(V+\mathrm{IR}s}{\mathrm{AKTC}N_{\mathrm{Se}}}-1]$$

### 2.2 Effect of PSC on the solar system and location of MEP

**Figure 3:**
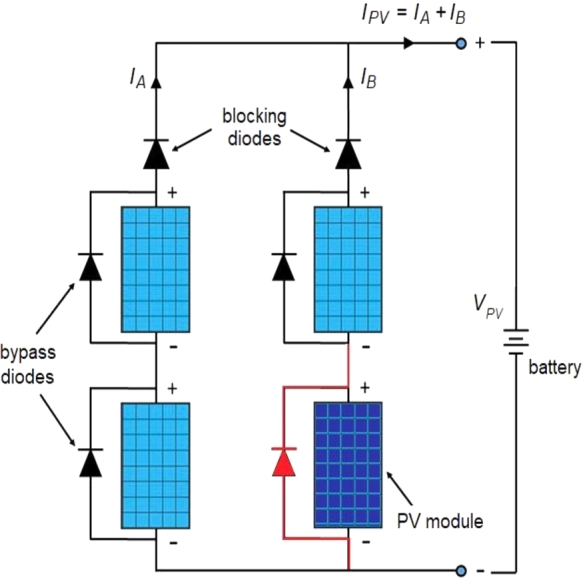
Configuration of a photovoltaic array with bypass and blocking diodes.

When three photovoltaic modules are connected in series means that there are three values of MEP along the P–V curve under PSCs. Likewise, if six PV modules connected in a row could have six maximum values. If all the solar cells in a photovoltaic array are identical under uniform solar irradiance consists of only one MEP. As shown in Fig. 3, the bypass diode is connected in shunt with every photovoltaic module to bypass the current around the module, so that the solar cells can continue to supply power even though the voltage is low.

Under uniform conditions of solar radiation, each photovoltaic cell is forward biased while the bypass diodes are reverse biased. Due to PSC, when the photovoltaic cells are reverse biased, bypass diode starts to conduct, so that current can flow into the external circuit through good solar cells. Under shaded conditions, the blocking diodes are connected in series with the photovoltaic modules to block the current from flowing back into them when voltage generated by photovoltaic modules is lower than that of the battery. Bypass diodes and blocking diodes can also be used to prevent the cells from self-heating under PSC.

**Figure 4:**
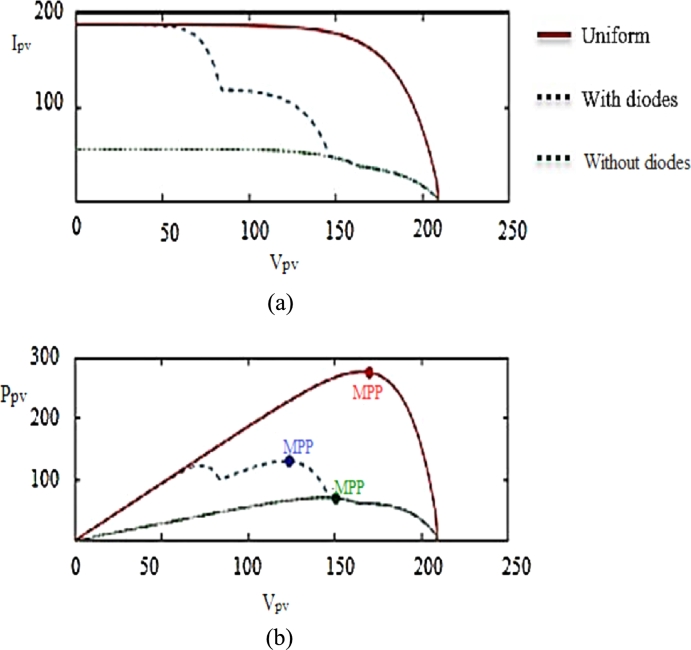
(a) Influence of bypass diodes on I–V characteristics. (b) Influence of bypass diodes on *P*–*V* characteristics.

Fig. 4 explains I–V and P–V characteristics of the photovoltaic system under constant radiation; under PSC without diodes, and under PSC with bypass and blocking diodes. Note that the presence of bypass diodes allows non-shaded photovoltaic modules to generate the maximal amount of current at a certain degree of solar radiation. In the absence of bypass diodes, the shaded modules limit the output power from the series string. Due to the effect of diodes, the PV curves have multiple local peaks and a global MEP (GMEP), as shown in the dotted line in Fig. 4. The presence of several maximum values in the P–V characteristics is a critical problem that most of the traditional MPPT methods can't solve.

Many conventional MPPT techniques start searching for the chosen area of PV curve; if that area is close to the local maximum, these methods can't find the GMEP, because they stop searching immediately after reaching the local maximum. In this case, the traditional MPPT method with a local search is not suitable for photovoltaic arrays with PSC. In addition, environmental conditions, namely temperature, shadows, and solar radiation changes affect the photovoltaic output. The DRLAMPPT is suggested in this article. It can be applied to photovoltaic systems to identify the clear difference between the GMEP and a local MEP. Eight photovoltaic modules connected in series are used for the experimental prototype and MATLAB simulation.

Solar energy has non-linear properties and its behavior is remarkably influenced by the changes in radiation and temperature. There will be only single optimal terminal voltage of the photovoltaic field that enables the photovoltaic module to operate in the MEP under certain climatic conditions. Therefore, it is necessary to develop an intelligent MPPT controller to extract MPP at any time and to conquer the limitations of conventional algorithms. There are several peaks in the PV curve of a photovoltaic module among PSCs. Therefore, an intelligent MPPT controller should be considered to conquer the limitations of conventional techniques. Fig. 1 represents the schematic outline of the solar power plant with photovoltaic array, DC/DC step-up converter, 3-*ϕ* 2 level PWM-VSI, MPPT controller, CCC, and 3-*ϕ* traditional power grid. A photovoltaic array with DC/DC converter can control the voltage with the variations in duty cycle *D* [23] is given by Eq. (7).

(7) $$D=-1-\frac{V_{m}}{V_{\mathrm{out}}}$$

This work proposes DRLA to drive the MPPT controller, including DDPG. The working principle of this algorithm will be introduced in the next chapter.

## 3 DRLA for MPPT control

To resolve the continuous MPPT problem of a solar photovoltaic system a model free RL technique is proposed in this work. The RL method allows studying the performance of the system based on the reaction of the interaction with the photovoltaic source. The development of RL algorithm for photovoltaic system operation should be based on the Markov decision problem (MDP).

### 3.1 General model of RL

RL is a type of unsupervised learning method, based on the neutral stimuli and reaction between the representative and its interactive environment [34]. RL is used to define the behavioral policies or strategies to maximize the overall benefit expected from trial-and-error interactions with the environment. Generally, RLA includes representative, environment, action, state, and benefit. Then the environment is the object of the representative, and the representative refers to the RLA. The environment will begin with sending a state, and the representative will react or take action depends upon his/her ability.

Then the representative receives benefits and next state from the environment, then updates the knowledge with the benefits to assess the previous action. When the environment sends the final state, this episode ends and another episode begins. This loop continues until the specified criteria are reached [31]. To determine the best strategy, few techniques use the value function $V^{\pi }(x)$ [26] is given by Eq. (9), which determines the extent to which the representative can reach the given state. It is the predicted benefit if the strategy ‘*π*’ of state ‘*x*’ is followed. Some other techniques are depending upon the action-value function $Q^{\pi }(x,a)$ [26] represents the predicted benefit of current state ‘*x*’ by taking the action ‘*a*’ under the policy ‘Π’. This action- value function is given by Eq. (8)

(8) $$Q^{\pi }(s_{t},a_{t})=E[R_{t}|x_{t}=x,a_{t}=a]==E\{\sum_{t=0}^{t=n}{ϒ}^{k}r_{t+k+1}/x_{y}=x\},$$

(9) $$V^{\Pi }(x)=E\{R_{t}/x_{y}=x\}=E\{\sum_{t=0}^{t=n}{ϒ}^{k}r_{t+k+1}/x_{y}=x\}$$

*Q* Learning is an off-policy RLA is becoming attractive in different areas. In *Q* learning, the function Q(s,a) may be expressed in an iterative form as per the Eq. (10) [26].

(10) $$Q^{\Pi }(x_{t},a_{t})=E[r_{t+1}+\gamma Q^{\pi }(s_{t+1},a_{t+1})/x_{y},a_{t}]$$

In the long term, largest total benefit is attained through the best strategy $\Pi^{⁎}$ given by Eq. (11). The optimum value function and action-value function [26] are given by Eq. (12) & Eq. (13).

(11) $$\Pi^{⁎}=\mathrm{argmax}V^{\Pi }(x)$$

(12) $$V^{⁎}(x)=\max V^{\Pi }(x)$$

(13) $$Q^{⁎}(x,a)=\max Q^{\Pi }(x,a)$$

One of the major challenges faced by RLA was dealing with the continuous action spaces. If the action space is excessively discrete, it creates a problem with spatial properties. However, inadequate discretization of the action space will ignore valuable information about the geometry of the action space. Therefore, the RLA is limited to a compact and discretized grid environment, making it less suitable for most dynamic systems. One of the most interesting areas of modern artificial intelligence is DRLA, which allows the representative to learn independently based on the results of interaction with a particular environment. DRLA is the integration of DLA and RLA, in which a representative is based on the interacting results with a particular environment, has achieved great success in different areas, such as games, power systems, digital image processing, etc. RLA uses a *Q* table for storage, index, which is sometimes not possible in practical applications with large state and action spaces is a major disadvantage. Therefore, ANN can be applied to estimate the value function or policy function.

### 3.2 DRLA

**Figure 5:**
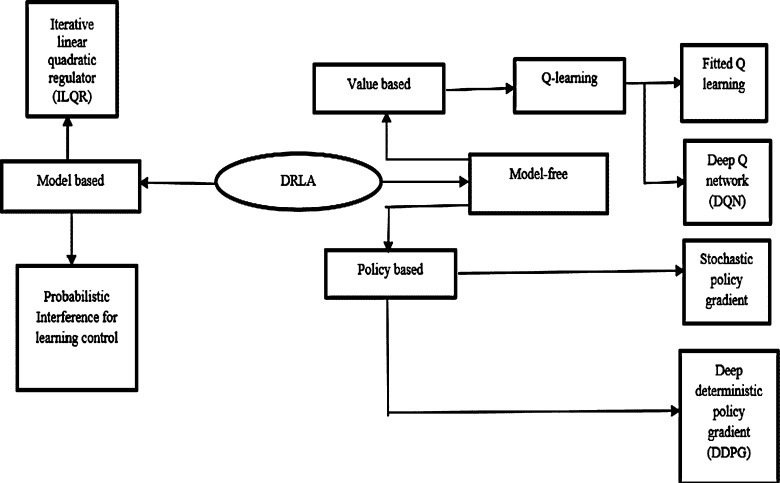
Introduction to DRLA.

There are two types of techniques, which include model based, & model free is represented in Fig. 5. In the model based DRLA the structure is already known and trained. One of the great advantages of model based DRLA; it needs multiple samples to train. Model-free RL is more profitable, the exact description of the environment does not need to be efficient, and it is not computationally complicated. Model-free DRLA is classified into value based and strategy based. The value-based attempts to improve the value function through each iteration until the occurrence is reached. Following is the objective function and updated value [31]. They are given by Eq. (14) & Eq. (15).

(14) J(θ)=E⌊(rt+1+γmax⁡Q(xt+1,πat+1|θ)−Q(xt,at|θ)⌋2

(15) θt+1=θt+α⌊(rt+1+γmax⁡Q(xt+1,aat+1|θ)−Q(xt,at|θ)⌋2∇θQ(xt,at|θ)

*α* is rate of learning,

*θ* represents weights of NN.

The strategy-based method directly optimizes the quantity of interest by updating the strategy at each time and determines the new strategy till the strategy converges. The objectives function [31] is given by Eq. (16).

(16) ∇θJ(θ)=E[∑t=0T∇θlog⁡πθ(at|xt)∑t=0Tr(xt,at)]

### 3.3 MDP model of a photovoltaic system

To develop DRLAMPPT of a system, it is necessary to define the MDP model of a given photovoltaic system Generally, MDP is represented with the state (*X*), action (*A*), target (*T*), benefit (*R*). Where *X* is the set of states that explains all the operating points of the photovoltaic system while *R* is the reward or benefit function, which is the amount of immediate benefit expected when taking an action from the present state. They are given by Eq. (17)

(17) $$P_{xx^{\prime }}^{a}=P[X_{t+1}=X^{\prime }|X_{t}=x,A_{t}=a]$$

Where *A* is a finite set of actions, i.e., the change in duty cycle applied to the DC-DC converter to change the operating point of the photovoltaic system. *T* is the transition function. The representative develops a strategy and trains how to get the maximum benefit for completing an episode. Therefore, we strengthen (reinforce) the representative with positive benefits to choose the right action and negative benefits for bad performances. To implement DRLAMPPT control predefined states, action spaces, and benefits must be calculated and derived [31]. They are given by Eq. (18) & Eq. (19)

(18) $$R_{x}^{a}=E[R_{t+1}|X_{t}=x,A_{t}=a]$$

(19) $$X=\{V_{\mathrm{pv}},I_{\mathrm{pv}},D,\Delta D\}$$

The action spaces are the perturbation of duty cycle, which includes negative, positive and unchanged. These action spaces are given by Eq. (20).

(20) A={set of actions (a)|+ΔD,0,−ΔD)

As per Eq. (21), benefit function [32] is represented as:

(21) $$r=r_{1}+r_{2}+r_{3}$$

(22) $$r_{1}=\frac{P_{t+1}}{P_{\mathrm{MEP}.\mathrm{STC}}}$$

(23) $$r_{2}=\begin{array}{cc} {\left(\frac{P_{t+1}}{P_{\mathrm{MEP}.\mathrm{STC}}}\right)}^{2}\; & \text{if}\;\Delta P\geq -\delta_{1}\\ 0\; & \text{if }\Delta P<-\delta_{1} \end{array}$$

(24) $$r_{3}=\begin{array}{cc} 0\; & \text{if }0\leq D\leq 1\\ -1\; & \text{otherwise} \end{array}$$

(25) $$\Delta P=P_{t+1}-P_{t}$$

where $\Delta \delta_{1}$ is considered as the small area (small number) around the MEP and is used to prevent the $P_{\mathrm{MPP},\mathrm{STC}}$ (MPP at standard test conditions). This proposed work benefit function comprises three components. First, $r_{1}$ is the benefit obtained at each time step in the given episode given by Eq. (22); it helps the representative to differentiate between the local MEPs and GMEP, higher benefits obtained by the representative if he stays at GMEP continuously. Second, according to the value of $r_{2}$ represented by Eq. (23), the representative receives a positive benefit when power increases; otherwise, zero benefits. As per the value of $r_{3}$ given by Eq. (24), if the duty cycle (*D*) is out of the boundary, then the representative receives a penalty. Δ*P* is considered to be change in the power given by Eq. (25).

### 3.4 DDPG based DRLAMPPT control

**Figure 6:**
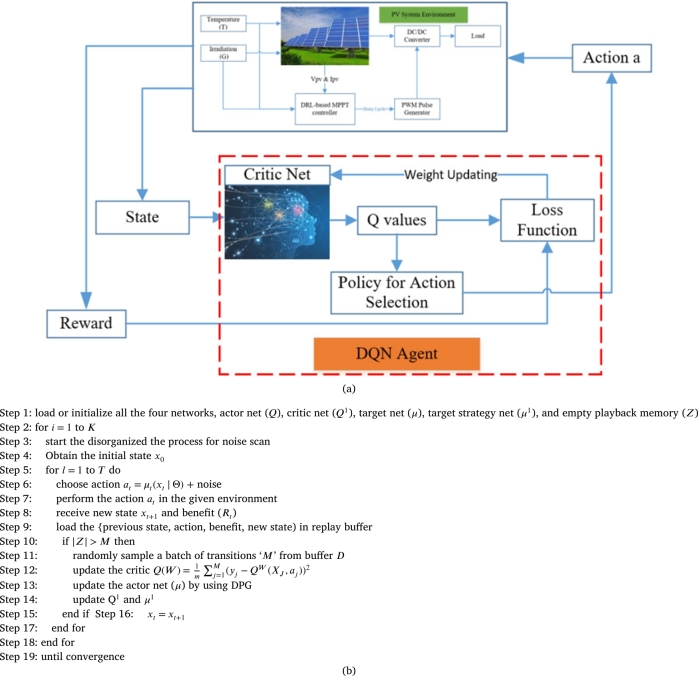
(a) Diagram of DDPG 6(b) Deep Deterministic Policy Gradient Algorithm for MPPT.

In this section, the formulation of DDPG based DRLA is explained as shown in Fig. 6(a). DDPG is an off-strategy algorithm, which handles continuous action spaces, so it is suitable for task management than DQN, which only deals with discrete action spaces. Compared with the value-based method, the strategy gradient method directly optimizes the strategy and selects the actions based on it. DDPG comprises four neural networks (NN) represented in Fig. 4. The first one is an actor net (*μ*) provides state-based actions; second one is critic net (*Q*) anticipates whether the action is good or bad for the given state. Third and fourth networks are target actor net ($\mu^{1}$) target strategy net ($Q^{1}$) stabilizes the process of learning. Actor and critic NN have a similar structure but with different weights. The deep deterministic policy gradient algorithm for MPPT is shown in Fig. 6(b).

## 4 Controller for 3-Φ 2 level PWM-VSI for interfacing the conventional power grid

**Figure 7:**
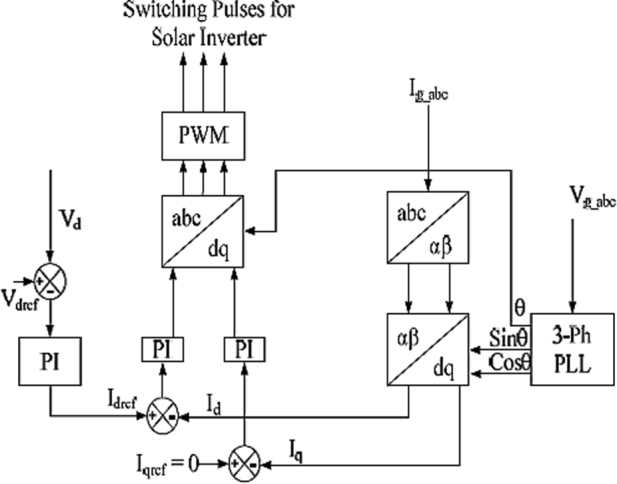
Representation of CCC.

Control of the 3-Φ 2 level PWM-VSI is provided through the CCC. A 3-Φ 2 level PWM-VSI is provided for interfacing the utility conventional power grid and solar PV array. This solar inverter converts the variable DC voltage of solar photovoltaic panels into an alternating voltage that can be fed to the conventional power grid. In this proposed work, a constant current controller along with 3-Φ PLL is provided to control the solar inverter. PLL is provided to track the phase voltages and frequency of the conventional power grid and also gives details regarding variations in the frequency of the utility conventional power grid. Detailed Constant Current Controller block diagram is shown in Fig. 7, which generates controlled pulses for the solar inverter. 3-Φ Conventional power Grid Voltage $Vg_{\mathrm{abc}}$ is sensed by using a voltage sensor and gives information to the PLL. To simplify the control and design process of 3-*ϕ* conventional power grid connected clarke's transformation can be used. Conventional power grid current $Ig_{\mathrm{abc}}$ is converted into *αβ* variables using Clarke's transformation

Clarke's transformation [41] can be obtained with the equations given by Eq. (26) & Eq. (27)

(26) $$I_{\propto }=\frac{2}{3}(I_{\mathrm{ga}})-\frac{1}{3}(I_{\mathrm{gb}}-I_{\mathrm{gc}})$$

(27) $$I_{\beta }=\frac{2}{\sqrt{3}}(I_{\mathrm{gb}}-I_{\mathrm{gc}})$$

Further *αβ* current variables can be converted into *dq* variables using park's transformation. Currents $I_{d}$ and $I_{q}$ are compared with the reference values $I_{\mathrm{dref}}$ & $I_{\mathrm{qref}}$ for processing the proportional-integral Controller to reduce the errors. Output signals of proportional-integral controllers are transformed into 3-Φ abc signals using inverse Park's transformation. These signals are compared with triangular waveforms to generate the pulses to the solar inverter.

## 5 Simulation & experimental results of the proposed DDPG based DRLA with CCC

In this work, simulation and experimental tests are carried out at standard temperature T=30°C and the sun irradiance has been varied from 1000 W/m^2^ to 500 W/m^2^. A PV string consists of 8 PV modules connected in series or in a row and parallel connected modules are not used in this simulation and experimental setup to test the proposed DRLAMPPT under PSCs.

In the simulation and experimental studies, 8 photovoltaic modules are connected in series to create the situations of partial shading. Every solar photovoltaic module comprises a bypass diode; hence 8 solar modules connected in series have 8 maximum values when all the 8 modules are at different solar irradiant conditions. In this simulation study, 4 PV modules are at the same solar irradiances of 1 kW/m^2^ and remaining 4 PV modules are at different solar irradiance conditions of 1 kW/m^2^, 0.9 kW/m^2^, 0.7 kW/m^2^, & 0.5 kW/m^2^ and the P–V curve is shown in Fig. 9 & Fig. 17(a).

### 5.1 Simulation results & discussion

**Figure 8:**
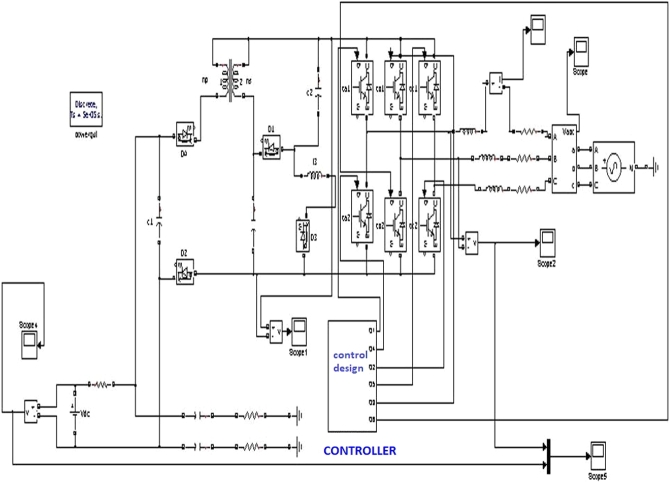
Simulation circuit illustrations of the suggested DRLA based partially shaded solar grid integrated system.

**Figure 9:**
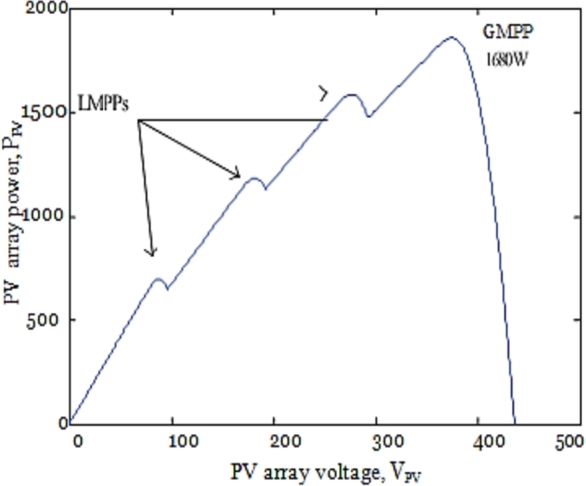
Simulation results of *P*–*V* characteristics.

**Figure 10:**
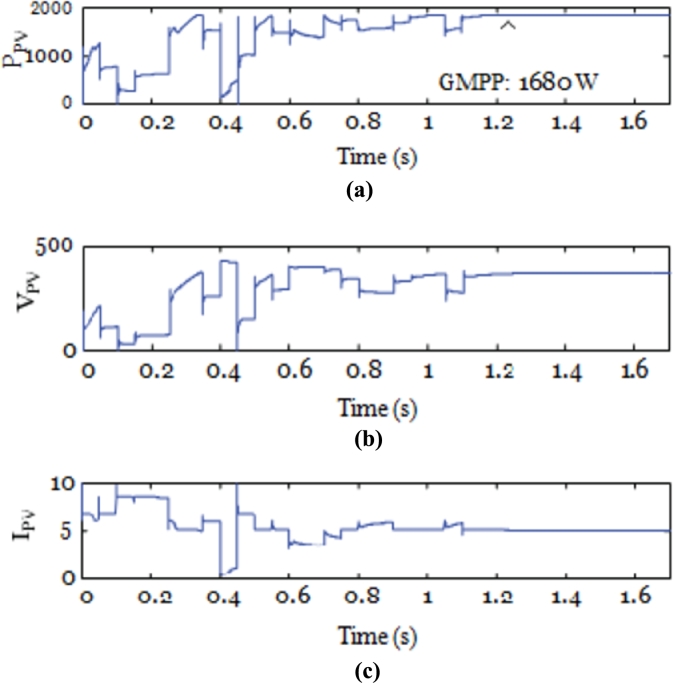
(a) power-time graph of photovoltaic system under PSC of 1 kW/m^2^, 0.7 kW/m^2^, and 0.5 kW/m^2^. (b) voltage-time graph of photovoltaic system under PSC of 1 kW/m^2^, 0.7 kW/m^2^, and 0.5 kW/m^2^. (c) current-time graph of photovoltaic system under PSC of 1 kW/m^2^, 0.7 kW/m^2^, and 0.5 kW/m^2^.

**Figure 11:**
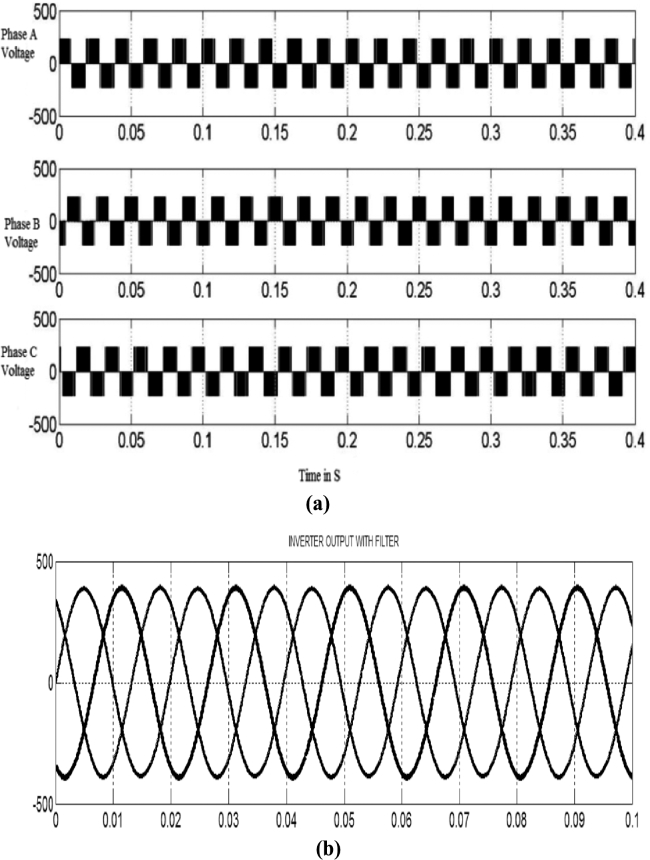
(a) 3-*ϕ* inverter output voltages without LC filter. (b) 3-*ϕ* inverter output voltages (line to line) with LC filter.

**Figure 12:**
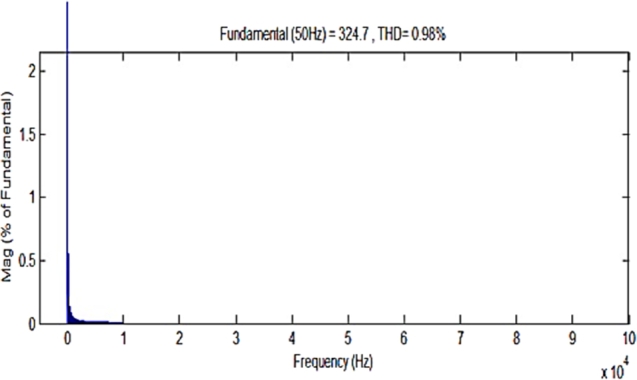
THD spectrum of 3-Φ inverter output voltage with LC filter.

To understand the behavior and overall performance of the suggested MPPT controller, a simulation study is conducted in MATLAB Simulink software using RL toolbox before the experimental investigation. A PV array which consists of 8 PV modules connected in series is considered. In this manuscript; a simulation model of a 2 kW solar power plant integrated with the conventional power grid is developed. The number of PV modules connected in series is 8 & the number of PV modules connected in parallel is 1. The solar photovoltaic array produces a variable DC output at different solar irradiances, and temperature level of PV array is 30 °C. Fig. 8 represents MATLAB simulation model of the 2 kW photovoltaic system comprises of photovoltaic array, MPPT controller, DC-DC converter, 3-*ϕ* 2 level PWM-VSI, &CCC incorporated with 3-*ϕ* conventional grid. The proposed algorithm is tested for different solar irradiance conditions of 1 kW/m^2^, 0.7 kW/m^2^, and 0.5 kW/m^2^, and PV characteristics of the photovoltaic system are shown in Fig. 9. Proposed DDPG based DRLAMPPT is developed in MATLAB to trace the GEMP of system and this MPPT controller begins with the initialization of duty cycle. Then the values of duty cycle keep on changing continuously and finally, it settles at 0.3 s as shown in Fig. 10. The output voltage of a solar photovoltaic system is low, even the series and parallel combination of solar photovoltaic cells do not produce the desired output. Hence DC-DC step-up converter is a circuit that produces output potential at a higher level than the input potential to provide the required output. Boosted DC Voltage is given to the 3-Φ 2 level PWM inverter. 3-phase LC Filter is provided at the output of inverter with an inductance of 5 mH & capacitance of 1000 μF. The combination of 3-Φ PWM-VSI & LC filter converts DC voltage into 3-Φ 440 V, 50 Hz pure sinusoidal AC with the reduced value of THD. Control of the 3-Φ 2 level PWM-VSI is provided through the CCC. 3-*ϕ* PWM-VSI inverter output voltage without and with LC filter is shown in Fig. 11. THD Spectrum of inverter output voltage with the combination of CCC & LC filter from MATLAB Simulink is shown in Fig. 12.

### 5.2 Experimental results & discussion

**Figure 13:**
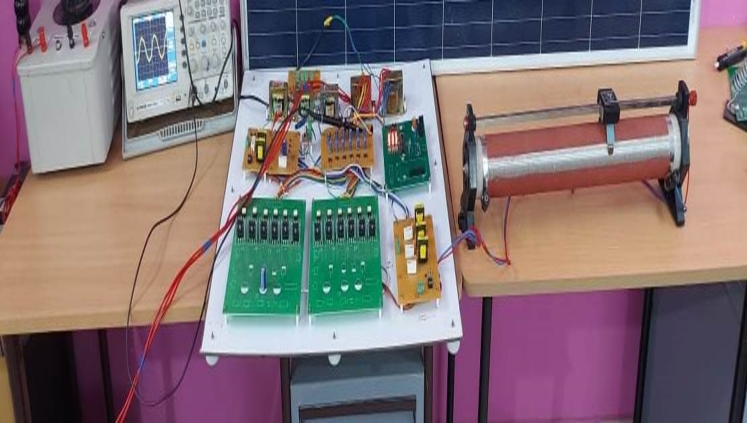
Experimental setup for the proposed conventional power grid connected DRLA based partially shaded PV System using CCC.

In this proposed work, to validate the simulation results an experimental prototype of the DRLA based shaded 2k W solar PV array integrated with a conventional power grid using CCC is developed & the experimental prototype is shown in Fig. 13. This experimental setup was designed and built with a PV array, DSPIC30F2010 processor, DSPIC30F connector board; traditional DC-DC step-up converter, a voltage sensor, a current sensor, and a LC filter. PV array comprises of 8 PV modules connected in series and power rating of each photovoltaic module is of 250 W. DSPIC30F2010 processor and connector board are used to produce the duty cycle (*D*) through the PWM pin and connector board also has analog to digital channels. The switching frequencies of the 3-*ϕ* 2 level PWM-VSI and DC-DC step-up converter were fixed at 15 kHz. The technical parameters that were taken into account when developing the experimental configuration are given in Table 2.

**Table 2:** Parameters considered in the design of prototype.

S.No.	Parameters	Value	Unit
1	Solar photovoltaic module rating	250	W
2	No. of photovoltaic modules connected in series	8	No.
3	No. of photovoltaic modules connected in parallel	1	No.
4	Maximum voltage rating of each module	37.25	V
5	Maximum current rating of each module	6.25	A
6	Filter inductance per phase	5	mH
7	Filter capacitance per phase	1000	μF
8	Grid RMS voltage (Line to Line)	440	V
9	Grid frequency	50	Hz

Each photovoltaic module is designed with silicon monocrystalline modules. The maximum voltage & current ratings of each photovoltaic module are 37.5 V & 6.25 A respectively. DC-DC step-up power electronic converter is constructed with an IGBT switch (CT60AM-18B, 900 V, 60 A, N - CHANNEL) together with a gate driver circuit (TLP250). The 3-Φ 2 level PWM-VSI is developed with six MOSFET switches (IRF840, N channel, 800 V, and 8 A).

This 3-Φ 2 level PWM-VSI receives a constant DC voltage of 600 V from the DC-DC step-up converter and produces an output voltage of 3-Φ 440 V, 50 Hz and is fed to the 3-*ϕ* conventional power grid. Hall Effect voltage and current sensors are provided near the conventional power grid to sense the 3-Φ voltages & currents and then fed to the 3-phase PLL (CD4046BK3). The proposed MPPT control and CCC are developed using DSPIC30F2010 processor. This processor provides the control to tune the MEP of the solar photovoltaic array using DDPG based DRLA, DC bus voltage regulation, control of 3-*ϕ* PWM-VSI, and system synchronization. Inverter Output voltages with and without LC filter are shown in Fig. 18, Fig. 19. In this section, various PSC will be applied to test and verify the proposed DDPGMPPT method. The experimentations were carried out under different irradiance conditions as described in MATLAB simulation. To validate efficiency of the suggested DDPG based DRLAMPPT, it is tested with different irradiance conditions of 1 kW/m^2^, 0.7 kW/m^2^, and 0.5 kW/m^2^.

**Figure 14:**
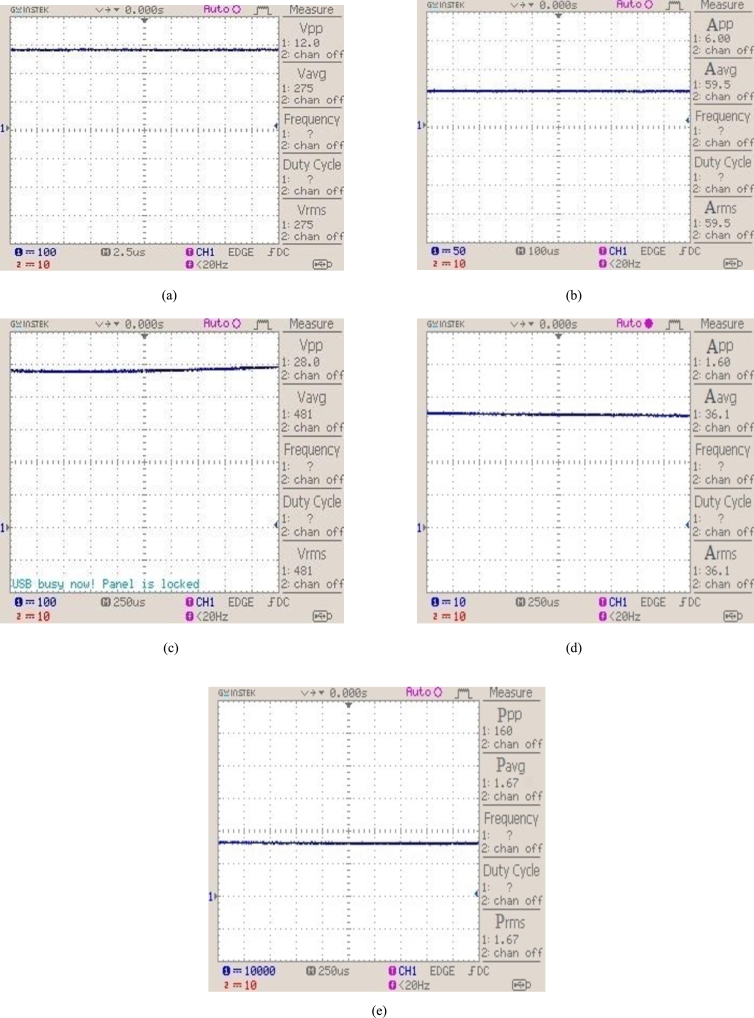
(a) 2 kW photovoltaic system output voltage with irradiance of 1 kW/m^2^. (b) 2 kW photovoltaic system output current with irradiance of 1 kW/m^2^. (c) DC/DC step-up converter output voltage. (d) DC/DC step-up converter output current. (e) Photovoltaic system power output with irradiance of 1 kW/m^2^.

**State 1**: Firstly, the experimental set up is tested with 1 kW/m^2^ irradiance and one photovoltaic module is shaded and voltage, current, and power are shown in Fig. 14.

**Figure 15:**
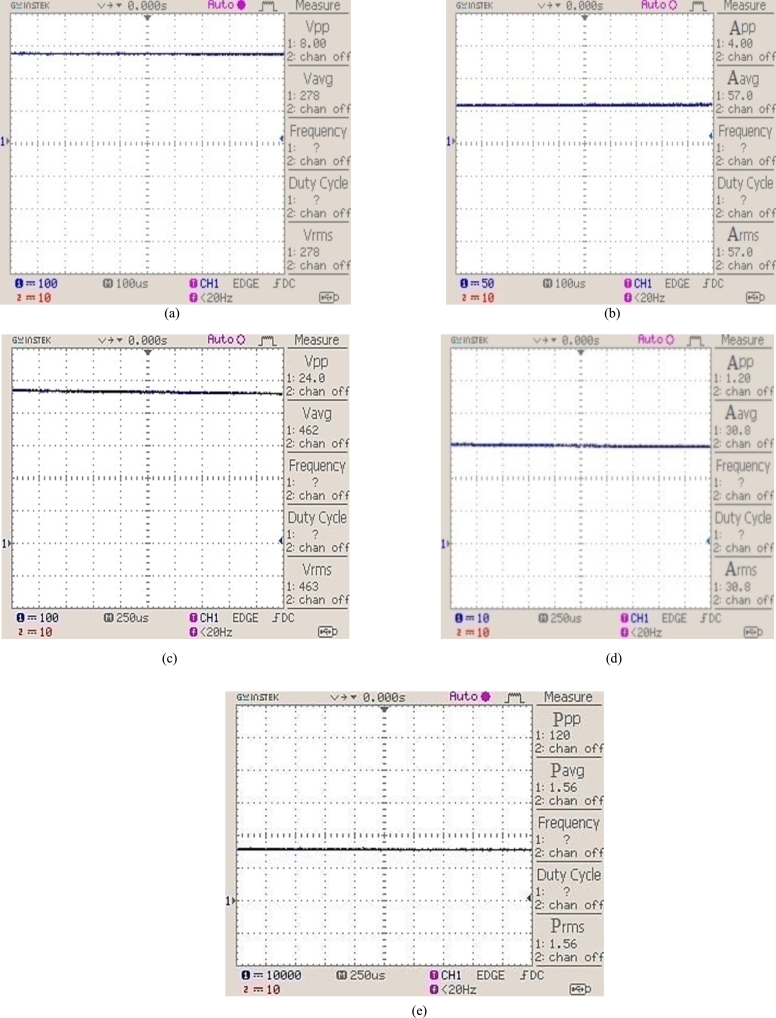
(a) 2 kW photovoltaic system output voltage with solar irradiance of 0.7 kW/m^2^. (b) 2 kW photovoltaic system output current with solar irradiance of 0.7 kW/m^2^. (c) DC/DC step-up converter output voltage. (d) DC/DC step-up converter output current. (e) Photovoltaic power output with irradiance of 0.7 kW/m^2^.

**State 2**: When two modules are shaded in the given system, then solar irradiance is reduced to 0.7 kW/m^2^. Fig. 15 shows voltage, current, and power waveforms.

**Figure 16:**
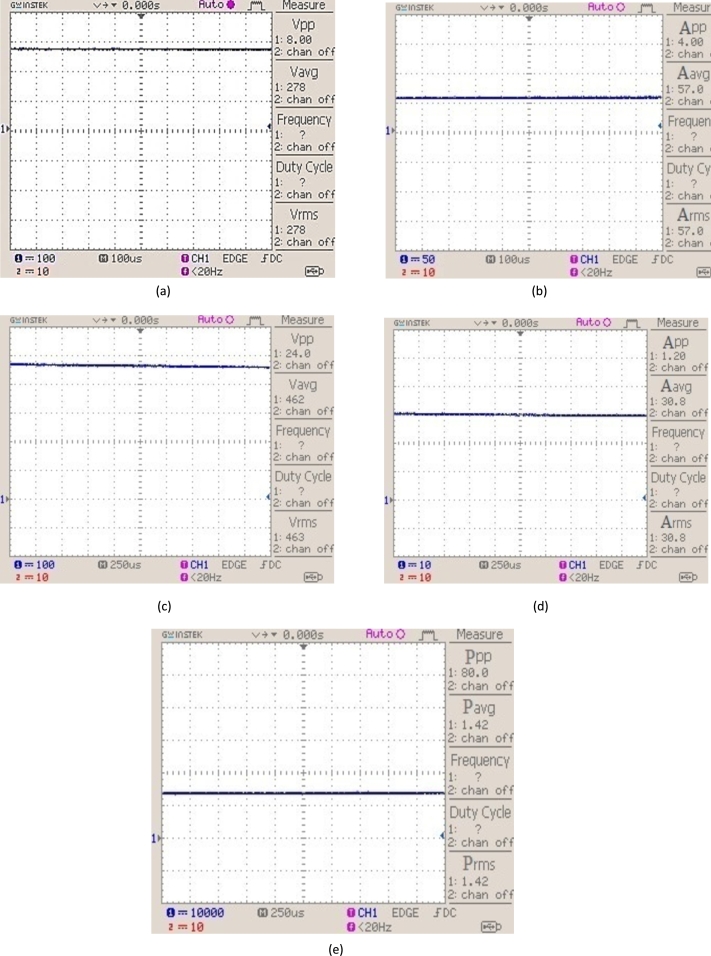
(a) 2 kW photovoltaic system output voltage with irradiance of 0.5 kW/m^2^. (b) 2 kW photovoltaic system output current with irradiance of 0.5 kW/m^2^. (c) DC/DC step-up converter output voltage. (d) DC/DC step-up converter output current. (e) Photovoltaic power output with irradiance of 0.5 kW/m^2^.

**State 3**: When four photovoltaic modules are shaded in the given photovoltaic system, solar irradiance is reduced to 0.5 kW/m^2^. Fig. 16 shows voltage, current, and power waveforms.

**Figure 17:**
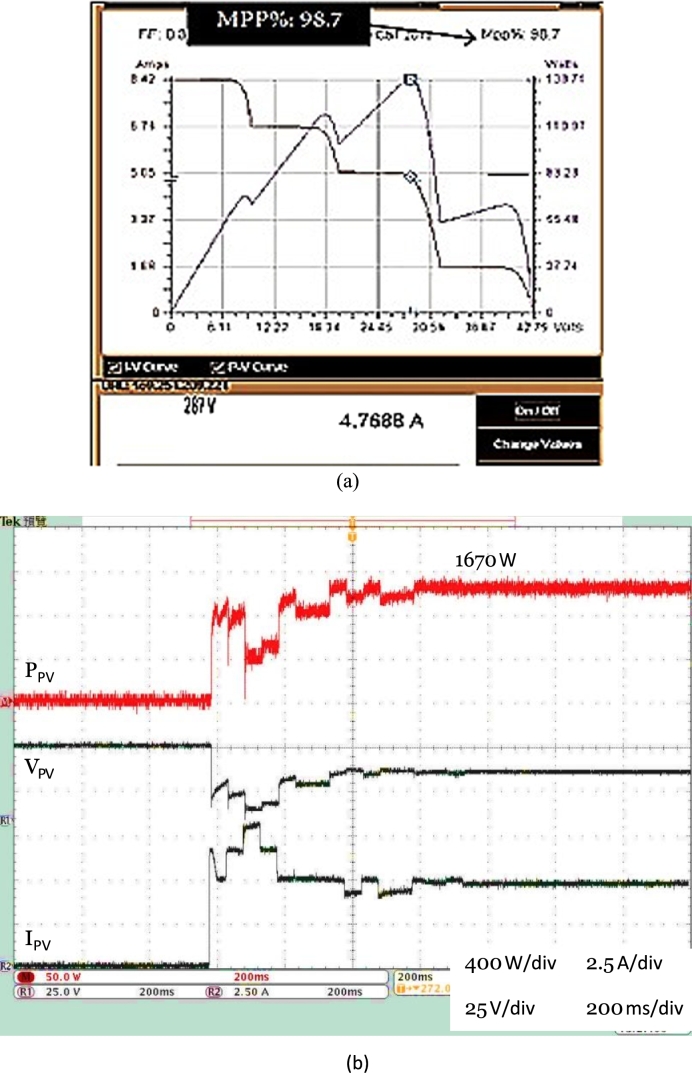
(a) VI and PV characteristics. (b) Hardware setup results of DDPG based DRLAMPPT under different irradiances.

The experimental tests were carried out under similar conditions as simulation. The change in the direction of the proposed algorithm is always aimed at the global MEP. Fig. 17 represents PV, IV characteristics and voltage, current, and power outputs of photovoltaic system under different irradiance conditions such as 1 kW/m^2^, 0.7 kW/m^2^, & 0.5 kW/m^2^. Proposed DRLA can track the GMEP and make sure that the photovoltaic system at 98.7% of maximum power and finished the search GMEP within 1 s.

**Figure 18:**
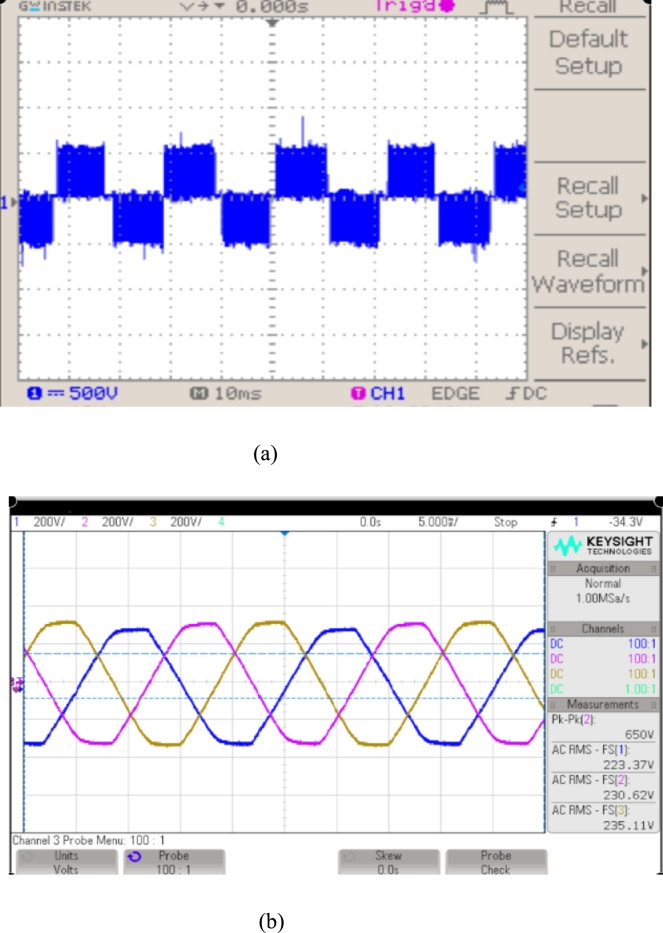
(a) 3-Φ 2 level PWM-VSI output voltage without LC filter. (b) 3-Φ 2 level PWM-VSI output voltage with LC filter.

**Figure 19:**
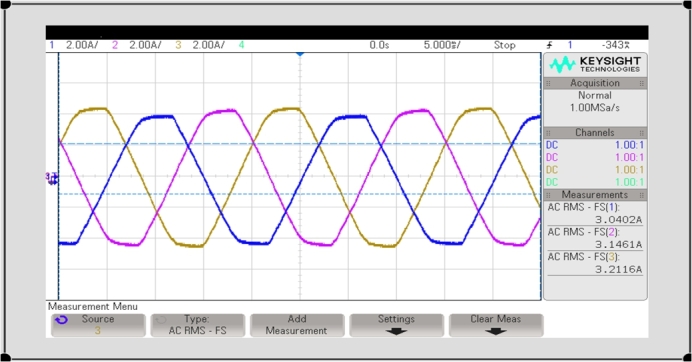
3-Φ 2 level PWM-VSI output current with LC filter.

**Figure 20:**
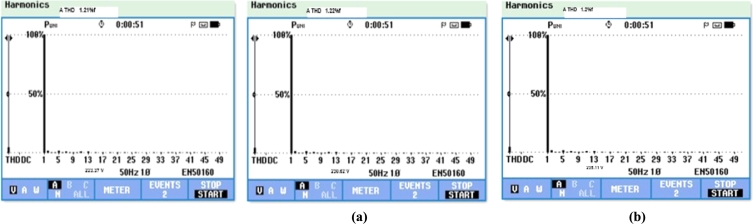
(a) THD spectrum of 3-Φ inverter output (phase-A) voltage with LC filter. (b) THD spectrum of 3-Φ inverter output (phase-B) voltage with LC filter. (c) THD spectrum of 3-Φ inverter output (phase-C) voltage with LC filter.

A 2 kW DRLA based partially shaded solar photovoltaic system is incorporated with 3-Φ conventional grid through DC-DC step-up converter, 3-Φ 2 level PWM-VSI. Control of 3-Φ 2 level PWM-VSI is maintained through CCC. This follows the conventional power grid phase and frequency. Using CCC, 3-Φ PWM-VSI output voltages & traditional power grid voltages are in the same phase. Experimentally CCC together with low pass LC filter gets rid of the inverter output voltage harmonics and produces 3-Φ output voltages to the low value of THD i.e., 1.22%, 1.21%, & 1.2% are shown in Fig. 20. The comparisons between the simulation and the experimental results are given in Table 3.

**Table 3:** Comparison of simulation results & experimental results.

S.No.	Parameter	Simulation	Experimental
1	Inverter output voltage	230	227.4
	Phase to phase		
2	THD of Inverter output voltage with LC filter	0.98%	1.2%
3	Maximum power point at an irradiance of 1 kW/m^2^	1680 W (99%)	1650 W (98.7%)
4	Maximum power point at an irradiance of 0.7 kW/m^2^	1590 W (99%)	1520 W (98.8%)
5	Maximum power point at an irradiance of 0.5 kW/m^2^	1470 W (98.9%)	1420 W (98.5%)

## 6 Conclusion

In addition to the development of photovoltaic cell materials to effectively improve the energy conversion, it is also necessary to design and develop new MPPT methods, such that these methods can extricate accurate value of GMEP at different irradiances, such as PSC. This manuscript presented a new DDPG based DRLA to track the GMEP under PSC with different irradiance conditions of 1 kW/m^2^, 0.7 kW/m^2^, & 0.5 kW/m^2^. This DDPG based DRLAMPPT controller is designed with ANN to estimate the strategy functions of photovoltaic environment rather than using lookup tables, so this proposed MPPT controller eliminates the larger memory requirements. Here the environment is solar PV model; the representative is DRLA, and while the action is change of duty cycle. This technique starts by sending the preceding state to representative, depends upon its knowledge takes reaction to the preceding state. Then the PV environment gives a reward and succeeding state. Then the representative can learn to act based on the present state and benefit received from the PV environment. DRLAMPPT continuously adopts the change in climatic conditions of the solar system, adjusts the duty cycle as well as operating potential of the PV system and attains the GMEP with in less time of computation i.e. 0.8 s during PSCs. Compare with the conventional MPPT techniques; this method can trace the GMEP with remarkable speed of convergence, better efficiency, and also reduces the randomness in convergence. Suggested DDPG based DRLAMPPT automatically adjusts the duty cycle to extricate the GMEP during change in climatic conditions. In this proposed work, an experimental prototype of 2 kW solar photovoltaic power plant which comprises a photovoltaic array, DRLAMPPT controller, step-up power electronic DC-DC converter, 3-Φ PWM-VSI is incorporated with conventional power grid using CCC (Constant Current controller) is developed using DSPIC30F2010. The conventional power grid-tied 2 kW solar photovoltaic system modeling and simulation is also done using MATLAB Simulink A 3-Φ 2 level PWM-VSI in conjunction with LC filer converts available DC voltage into 3-Φ, 440 V, 50 Hz Sinusoidal AC Voltage. Control of 3-Φ 2 level PWM-VSI is maintained through CCC. This follows the conventional power grid phase and frequency. Using CCC, 3-Φ PWM-VSI output voltages & traditional power grid voltages are in the same phase. Experimentally CCC together with low pass LC filter gets rid of the inverter output voltage harmonics and produces an output voltage to the low value of THD i.e. 1.1%. To validate the capability of the suggested MPPT technique both simulation & experimental outcomes are presented in this paper. The main con Hence DRLA based partially shaded solar photovoltaic sources can be incorporated with the traditional power grid by using CCC with the improved features of power quality. But the main disadvantage of CCC, without LC filter it is less efficient, produces lower power factor, and higher value of THD of inverter output voltages.

Complexity: Complexities involved in the implementation of DDPG are training of an agent takes more time because the training of both actor and critic networks are necessary. Behavior of actor network is highly dependent on the behavior of critic network it is very much necessary that both show adequate stable growth, which is very difficult to achieve.

Future work: In this study, to observe the performance of proposed DRLAMPPT simulation and experimental tests are conducted at different irradiance conditions and constant temperature. Suggested DRLAMPPT provides best efficiency and can track the GMEP within less time of computation. However, the main restriction of this work is that the suggested DRLAMPPT may not always detect the GMEP. Hence, further studies and research will be carried out at different irradiances and temperatures in the future to improve the traceability of DRLAMPPT.

## Declarations

### Author contribution statement

Radhika Guntupallia: Conceived and designed the experiments; Performed the experiments; Analyzed and interpreted the data; Contributed reagents, materials, analysis tools or data; Wrote the paper.

M. Sudhakaranb, P. Ajay-D-Vimal raj: Analyzed and interpreted the data; Contributed reagents, materials, analysis tools or data.

### Declaration of interests statement

The authors declare no conflict of interest.

### Data availability statement

Data will be made available on request.

### Funding statement

This research did not receive any specific grant from funding agencies in the public, commercial, or not-for-profit sectors.

### Additional information

No additional information is available for this paper.
